# Development and evaluation of the phenotypic 2G test to detect drug-resistant TB

**DOI:** 10.5588/ijtldopen.25.0326

**Published:** 2025-11-12

**Authors:** J.I. Garcia, E.T. Mambuque, A.D. Hicks, A. Schami, S. Munguambe, N. Gomez, G. Tembe, B. Saavedra, S.-H. Wang, J.-M. Balada-Llasat, B.I. Restrepo, M. Yotebieng, J. Gelfond, A.L. Garcia-Basteiro, J.B. Torrelles

**Affiliations:** 1 International Center for the Advancement of Research and Education (I•CARE), Texas Biomedical Research Institute, San Antonio, TX, USA;; 2 TB Group, Population Health Program, Texas Biomedical Research Institute, San Antonio, TX, USA;; 3 Centro de Investigação em Saúde de Manhiça (CISM), Maniça, Mozambique;; 4 Division of Infectious Diseases, Department of Internal Medicine, The Ohio State University, Global One Health initiative (GOHi), College of Medicine, Columbus, OH, USA;; 5 Department of Pathology, College of Medicine, The Ohio State University, Columbus, OH, USA;; 6 School of Public Health, University of Texas Health Science Center at Houston, Brownsville, TX, USA;; 7 South Texas Diabetes and Obesity Institute, School of Medicine, University of Texas Rio Grande Valley, Edinburg, TX, USA;; 8 Division of General Internal Medicine, Department of Medicine, Albert Einstein College of Medicine, The Bronx, NY, USA;; 9 Department of Population Health Sciences, UT Health San Antonio, San Antonio, TX, USA;; 10 Barcelona Institute for Global Health (ISGlobal), Hospital Clínic of Barcelona, Universitat de Barcelona, Barcelona, Spain;; 11 Centro de Investigación Biomédica en Red de Enfermedades Infecciosas CIBERINFEC, Barcelona, Spain.

**Keywords:** tuberculosis, Mozambique, DR-TB, drug susceptibility testing, *Mycobacterium tuberculosis*, thin-layer culture

## Abstract

**BACKGROUND:**

Early diagnosis of TB with drug susceptibility testing (DST) is critical to achieve successful treatment outcomes. We aimed to develop and test a novel colorimetric, 12-well, thin-layer agar-based test to assess its accuracy for TB diagnosis and DST in a clinical setting in Southern Mozambique.

**METHODS:**

Development of the first prototype of the second generation (2G) test in the laboratory setting followed by a cross-sectional diagnostic accuracy study with consecutive recruitment of subjects with microbiologically confirmed TB using GeneXpert MTB/RIF Ultra.

**RESULTS:**

In the laboratory setting, the 2G test showed 100% accuracy in detecting resistance of genotypically characterised drug-resistant *Mycobacterium tuberculosis* strains. In the clinical setting, the sensitivity of the 2G test to detect *M.tb* complex versus Xpert and Mycobacteria Growth Indicator Tube (MGIT) culture using fresh sputa was 45.9% and 45.2%, respectively. The 2G test sensitivity versus MGIT decreased to 23.1% when using frozen decontaminated sputum samples.

**CONCLUSION:**

In the clinical setting, the 2G test showed a low sensitivity versus Xpert and MGIT. The 2G test sensitivity was lower when frozen instead of fresh sputa was used. Despite these results, important information was collected to further improve this 2G test prototype and its implementation in resource-constrained settings.

TB caused by *Mycobacterium tuberculosis* (*M.tb*) is a leading infectious disease cause of death worldwide. Early TB diagnosis with drug susceptibility testing (DST) is critical to achieve successful treatment outcomes and is a key component of the End TB Strategy.^1^ DST is achieved via phenotypic or genotypic methods. Traditionally, phenotypic DST is performed on solid Löwenstein-Jensen or liquid media such as Mycobacteria Growth Indicator Tubes (MGITs) in a two-step process: a culture to identify *M.tb* growth and a sub-culture with the drugs to be tested. In addition to requiring biosafety level II-plus labs, the DST process, if available in resource-constrained settings, is tedious, requires skilled laboratory personnel, and is generally centralised in tertiary hospitals.^2^ Further, in some cases, it may take ≥3 months from sample collection to notification of results resulting in delays in proper treatment initiations, continued TB transmission, and higher mortality.^2–4^ Conversely, genotypic DST has several advantages, including a reduced time to result (<2 h for GeneXpert) and the possibility of deployment to point-of-care or near to point-of-care (POC/nPOC) settings.^5^ However, its widespread use in high-TB-burden settings is hindered by the need for regular power supply, equipment maintenance, lack of supplies (e.g., cartridges for Xpert), and, importantly, prohibited prices.^6^

Results from recent clinical trials have led to the recommendation to use a 6-month regimen consisting of bedaquiline (BDQ or B), pretomanid (Pa), and linezolid (LNZ or L) with or without moxifloxacin (MFX) to treat multidrug-resistant (MDR)- and rifampicin-resistant (RR)- or pre-extensively drug-resistant (pre-XDR) TB.^7–9^ Recent studies have highlighted the acquisition and transmission of BDQ-resistant strains and its direct implication in TB treatment failure.^10,11^ Therefore, scale-up of these new treatment options should be paired with proper DST capabilities with the drugs contained in the treatment regimens.^11^ The colorimetric thin-layer agar method is an alternative to classical phenotypic or genotypic DST methods, as evidenced by data from us and others with the colour plate agar-based culture test (TB-CX test; also known as colour test [CT], coloured agar-based culture test [CX test], or the first generation [1G] test).^12–17^ In initial field testing conducted by us across multiple settings and using freshly collected sputa, the 1G test identified drug-susceptible (DS)- and MDR-TB in ∼12 to 14 days with high sensitivity/specificity (>98%).

To expand DST capacities and incorporate new drugs included in the latest World Health Organization (WHO)-recommended regimens for DS- and DR-TB, we have developed the first prototype of the second generation (2G) test (see Figure 1). This 2G test is based on previous developments of the 1G test published elsewhere^12–18^ and contains 11 anti-TB drugs (Figure 1) at the critical concentrations recommended by the WHO for each specific drug^19^ (Supplementary Data). Here, we describe the development of the first 2G test prototype in the laboratory setting, as well as a proof-of-concept pilot study to evaluate its sensitivity for detection of *M.tb* and DST accuracy in a high-TB-burden setting in Southern Mozambique, where TB incidence is around 361 cases/100,000 people/year, and the estimated 12% HIV prevalence in adults fuels morbidity and mortality.^20,21^

**Figure 1.**
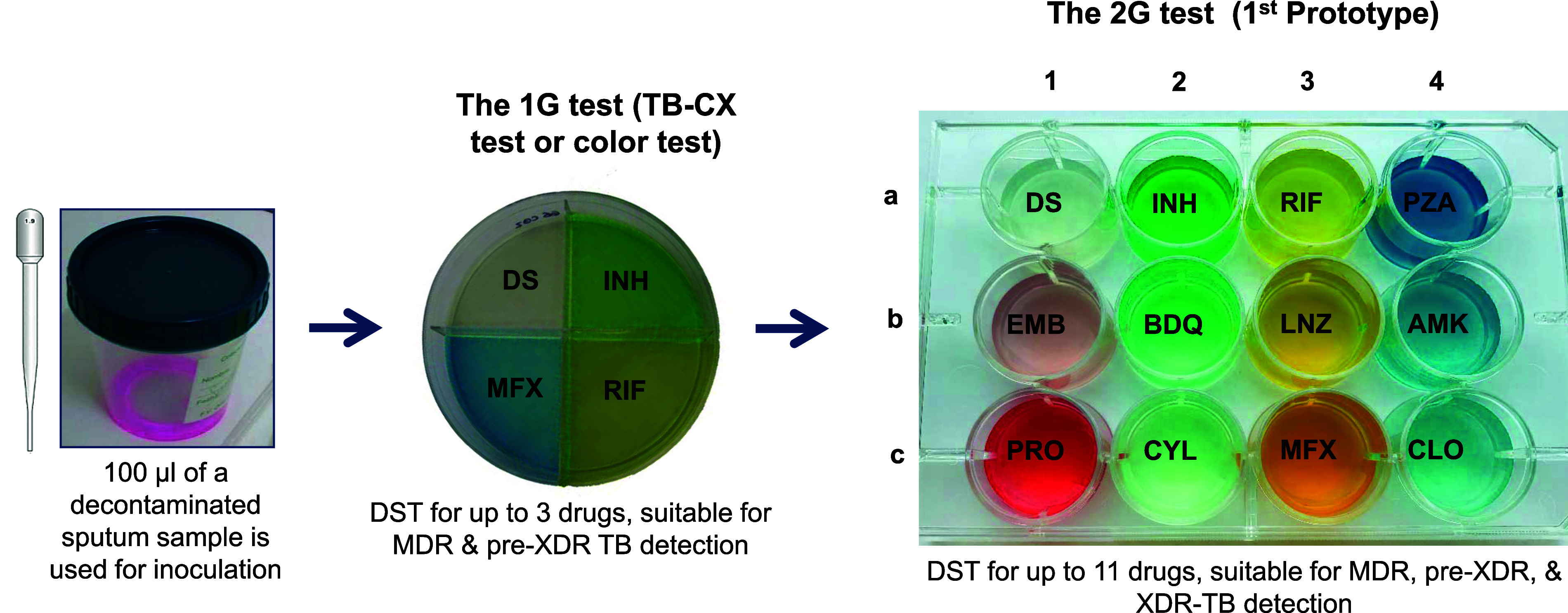
Evolution of the 1G test into the first prototype of the 2G test. Critical concentrations for each of the drugs are as follows: isoniazid (INH), 0.2 μg/mL; rifampicin (RIF), 1 μg/mL; pyrazinamide (PZA), 1,100 μg/mL; ethambutol (EMB), 7.5 μg/mL; bedaquiline (BDQ), 0.25 μg/mL; linezolid (LZD), 1 μg/mL; amikacin (AMK), 2 μg/mL; protionamide (PTO), 10 μg/mL; cycloserine (CYL), 32 μg/mL; moxifloxacin (MFX), 0.5 μg/mL; and clofazimine (CLO), 1 μg/mL. Drug-susceptible (DS) well without drug (control well).

## METHODS

Genotypically characterised drug-resistant *Mycobacterium tuberculosis* (DR-*M.tb*) strains (n = 21) with known resistance to isoniazid (INH), rifampicin (RIF), ethambutol, pyrazinamide, MFX, and amikacin (AMK) were used for 2G test development studies. For each *M.tb* strain, frozen stocks were freshly plated on 7H11 agar medium and recovered to prepare single bacterial suspensions to be used in the inoculation of the 2G test in all laboratory experiments presented.^22^ The 2G test follows the agar proportion method with the 1% critical proportion^23^ and has three possible results for the DS-well: positive (countable colonies), negative (no colonies), and uninterpretable (contamination, agar dehydration, or other reasons). For the drug-containing wells, there are three possible results: resistant (countable colonies), susceptible (no colonies), and uninterpretable (as defined for the DS-well). Days to a positive 2G test results for interpretation were measured for all 21 DR-*M.tb* strains under two storage settings: optimal storage conditions (at 4°C, ≤4-month period) and non-optimal storage conditions (room temperature, ≤4-month period). A detailed description of the 2G test preparation is provided in the Supplementary Data.

### Clinical study design in Manhiça, Southern Mozambique

This cross-sectional diagnostic accuracy study (under approval by the Mozambican National Bioethics Committee #423/CNBS/20) was performed at the *Centro de Investigação em Saúde de Manhica* (CISM), located in Manhiça, Southern Mozambique, from July 2021 to June 2023. Recruitment was conducted at the clinics of the Mozambican National TB Control Program, either in Xinavane Rural Hospital, Manhiça District Hospital, or Magude Rural Hospital. Participants with microbiologically confirmed TB using Xpert MTB/RIF Ultra (Cepheid, Sunnyvale, CA, USA) (Xpert Ultra),^24^ were consented and enrolled in the study (diagnostic Xpert Ultra). Participants underwent clinical assessments and provided a sputum spot sample in designated examination rooms to confirm the positivity of the initial Xpert result (confirmatory Xpert Ultra). This second sputum sample was the one used in this study and processed at the ISO 15189 accredited externally quality-assured BSL-3 TB laboratory at CISM. The index test (the 2G test) was compared to the reference standards MGIT culture or Xpert for diagnostic accuracy and MGIT DST for susceptibility testing accuracy. Assessors of the reference standards and the index test were not blinded. Study reporting followed Standards for Reporting Diagnostic Accuracy (STARD) guidelines.^25^ Generated datasets were kept at the CISM data centre. All the 2G test lots passed quality controls before being used at CISM.

### Sputum collection and processing at CISM

Xpert Ultra confirmed sputum samples were received refrigerated with ice packs at CISM within 12 h of sputa collection. These fresh sputa were decontaminated using the N-acetyl-l-cysteine–sodium hydroxide at 2% (NALC-NaOH) method and processed in parallel for liquid MGIT culture and the 2G test within 24–72 h of sample collection (fresh samples). When 2G tests were not available, fresh sputa were decontaminated and processed for MGIT, and an aliquot was stored at −80°C for later testing with the 2G test (frozen samples). A detailed description of the MGIT, MGIT DST, and 2G test processing at CISM is provided in the Supplementary Data.

### Data analysis

Percentages, median and interquartile range, and mean and standard deviation were used in descriptive statistics. Pearson’s χ² test and Student’s *t* test were used to determine associations between categorical and continuous variables. Accuracy measures such as sensitivity, specificity, and positive (PPV) and negative (NPV) predictive values and 95% confidence intervals (CI) were calculated. Stata 18 (StataCorp, College Station, Texas, USA) and GraphPad Prism vr. 10.0.0 (GraphPad Software, Boston, Massachusetts, USA, www.graphpad.com) were used for statistical analyses.

## RESULTS

**Figure 2.**
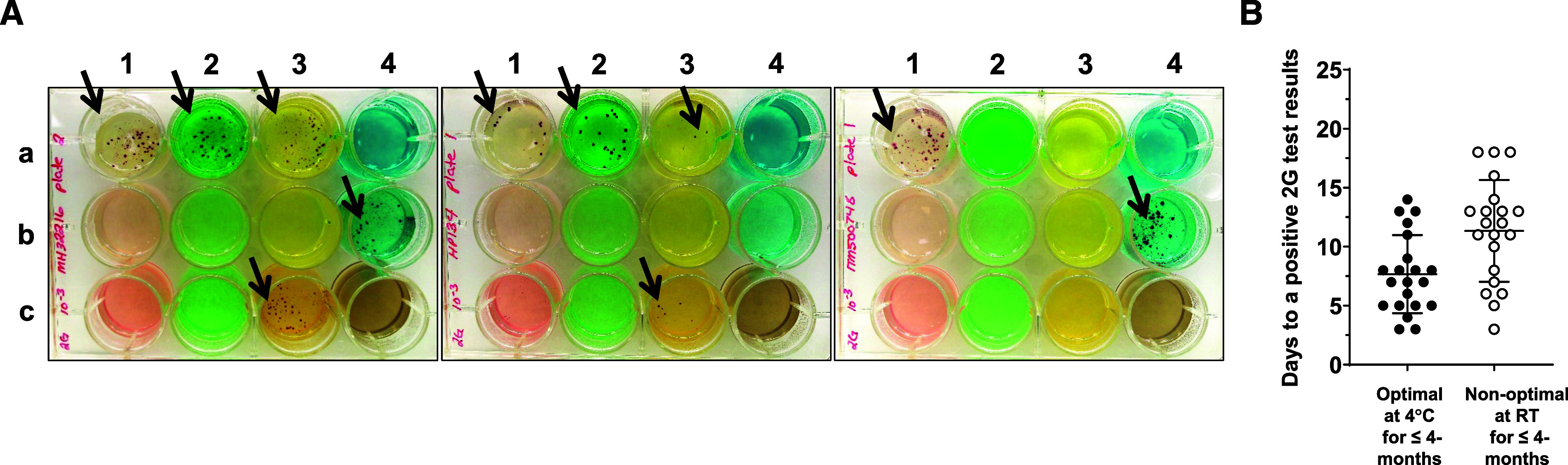
**A:** Results of the 2G test using different DR-*M.tb* strains in the laboratory setting. (Black arrows show *Mycobacterium tuberculosis* growth). Left plate: Growth of a DR-*M.tb* strain with resistance to INH, RIF, AMK, and MXF. Middle plate: Growth of a DR-*M.tb* strain with resistance to INH, RIF, and MXF. Right plate: Growth of a DR-*M.tb* strain with resistance to AMK. **B:** Days to a positive 2G test result using 21 DR-*M.tb* strains previously stored under optimal and non-optimal conditions.

Results of the 2G test DST for some of the 21 genotypically characterised DR-*M.tb* strains in the laboratory setting are shown in Figure 2A. 2G test DST showed 100% accuracy in detecting drug resistance to the DR-*M.tb* strains used when the 2G test was stored under optimal conditions (at 4°C) or non-optimal conditions (room temperature, ∼23°C to 30°C) for up to 4 months. Under optimal conditions, growth of the 21 DR-*M.tb* strains was detected at 7.67 ± 3.31 days (M ± SD; and interquartile range [IQR] 11 [5–10 days]), where the fastest growth was observed a soon as 3 days post-inoculation and the longest at 14 days (Figure 2B). Under non-optimal conditions, growth of the same 21 *M.tb* strains was detected at 11.33 ± 4.32 (M ± SD, and IQR 12 [7.5–18 days]), where the fastest growth was also observed at 3 days post-inoculation and the longest at 18 days (Figure 2B).

### Performance of the 2G test prototype in Manhiça, Southern Mozambique

**Figure 3.**
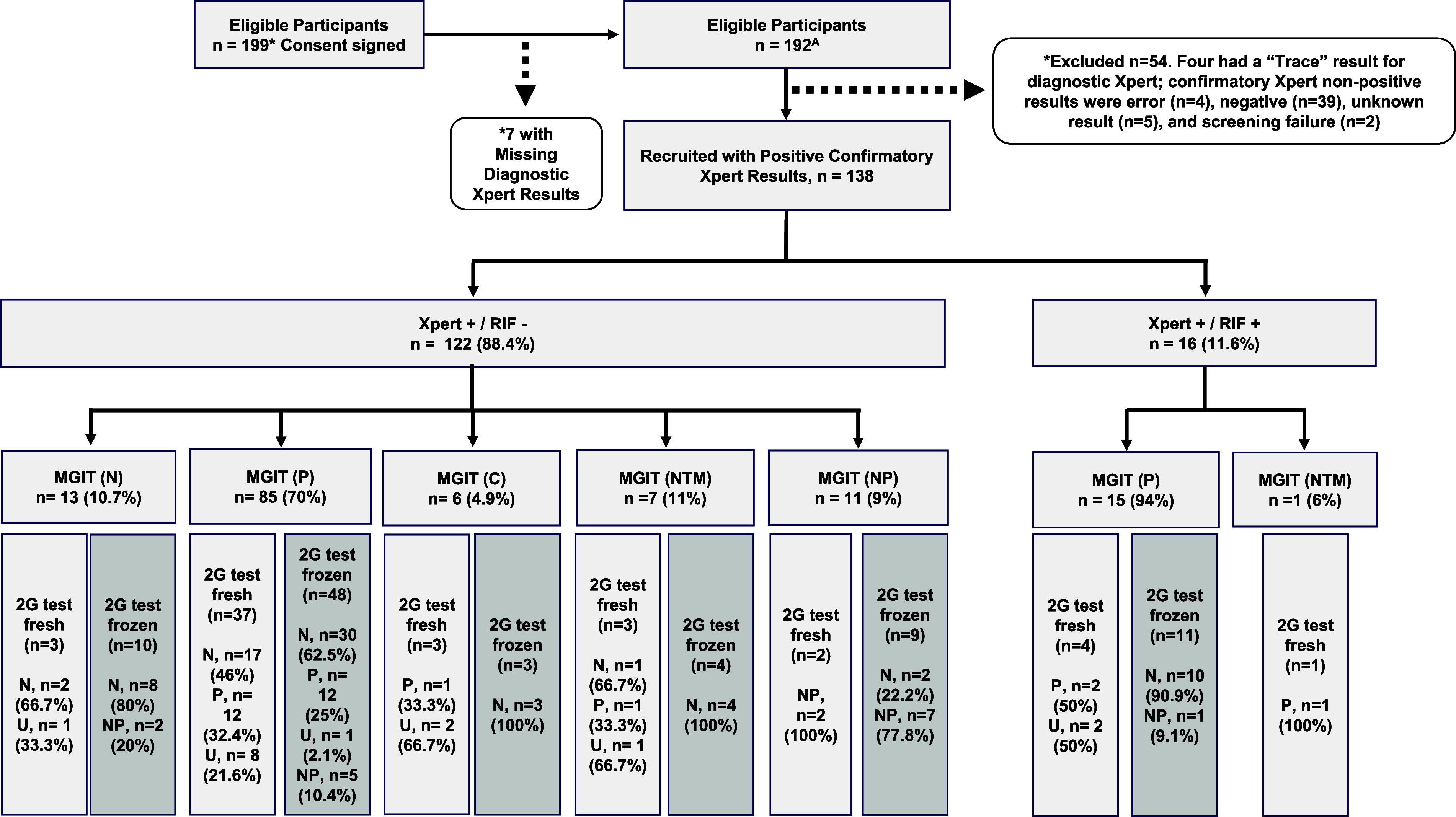
Study participants flow and tests performed using fresh and frozen sputa. ^**A**^Those with positive Xpert results at TB diagnosis. MGIT = Mycobacteria Growth Indicator Tube; P = positive; N = negative; C = contaminated; U = uninterpretable; NP = not performed; n = sample size.

The study recruitment flow is shown in Figure 3, and socio-demographic and clinical characteristics of all enrolled participants can be found in the Supplementary Data. Of the 199 participants who consented, 7 were excluded for having missing diagnostic Xpert results, and 54 were excluded for different reasons. Of those excluded, 39 (72.2%) were for having negative results on the confirmatory Xpert. This means that 39 of 177 (22.0%) participants with an initially positive Xpert in the routine specimen and not excluded for other reasons had a negative Xpert result in the second specimen (confirmatory Xpert), which was the one used for the study. The difference in days between obtaining the first sputum for primary Xpert diagnosis and the second sputum for confirmatory Xpert was 5.8 days ± 5.3 (M ± SD, and IQR 5 [2–8 days]). Of the 138 Xpert positive samples, 127 were also analysed by MGIT, showing 100 *M.tb* complex MGIT-positive results, 13 negative, 6 contaminated, and 8 non-TB mycobacteria (NTM). All sputum samples processed by MGIT culture were fresh (stored at 4°C for 24–72 h). Overall, of the 100 MGIT *M.tb* complex positive samples, 41% were processed simultaneously for the 2G test, and 59% were frozen for later 2G test processing. The MGIT contamination rate was at 4.7% (6/127), and the 2G test contamination rate was of 15.2% (7/46) and 1.4% (1/70) for fresh and frozen sputum samples, respectively.

### Diagnostic sensitivity of the 2G test versus Xpert using fresh/frozen sputum samples

For Xpert, we used fresh sputum samples; however, for the 2G test we used both fresh and frozen sputum samples. When using the same fresh samples (n = 37) for both tests, the sensitivity of the 2G test to diagnose TB versus Xpert was of 45.9% (95% CI: 29.5–63.1). However, when using frozen samples for the 2G test (n = 69), the sensitivity of the 2G test to diagnose TB versus Xpert decreased to 17.4% (95% CI: 9.3–28.4). Contaminated or uninterpretable results were excluded from the analysis. Differences in sensitivity of the 2G test compared to Xpert when using fresh (45.9%) compared to frozen (17.4%) samples were statistically significant (*P* value = 0.002). There were no differences in sensitivity by HIV status of participants.

### Diagnostic accuracy of the 2G test versus MGIT culture using fresh/frozen sputum samples

The sensitivity, specificity, PPV, and NPV of the 2G test to diagnose TB versus MGIT culture are shown in Table. Ninety-three 2G test and MGIT samples had valid results for accuracy analysis.

**Table.** Diagnostic accuracy of the second generation (2G) test to detect *Mycobacterium tuberculosis* complex versus Mycobacteria Growth Indicator Tube (MGIT)^**A**^ culture (n = 93).

	Sensitivity % (95% CI)		Specificity % (95% CI)		PPV	NPV
	Positive/N		Negative/N		% (95% CI)	% (95% CI)
All	26/83	31.3 (21.6–42.4)	10/10	100 (69.2–100)	100 (86.8–100)	14.9 (7.7–25.7)
2G fresh samples	14/31	45.2 (27.3–64)	2/2	100 (15.8–100)	100 (76.8–100)	10.5 (1.3–33.1)
2G frozen samples	12/52	23.1 (12.5–36.8)	8/8	100 (63.1–100)	100 (73.5–100)	16.7 (7.5–30.2)

A All MGIT sputa were processed fresh.

Differences in sensitivity of the 2G test compared to MGIT culture when using fresh samples (45.2%) versus frozen samples (23.1%) were statistically significant (*P* value = 0.036). Overall, there were a total of 26 positive results for both the MGIT and the 2G test. Of these 26, median time to a positive 2G test result in the 14 fresh samples was of 18.5 days IQR (12–20), and it was of 22.5 days IQR (19–33.5) in the 12 frozen samples studied. The 8.9 days difference in mean days to a positive result for frozen (26.3 days) compared to fresh (17.4 days) samples was statistically significant (*P* value = 0.004).

### 2G test DST and MGIT DST

**Figure 4.**
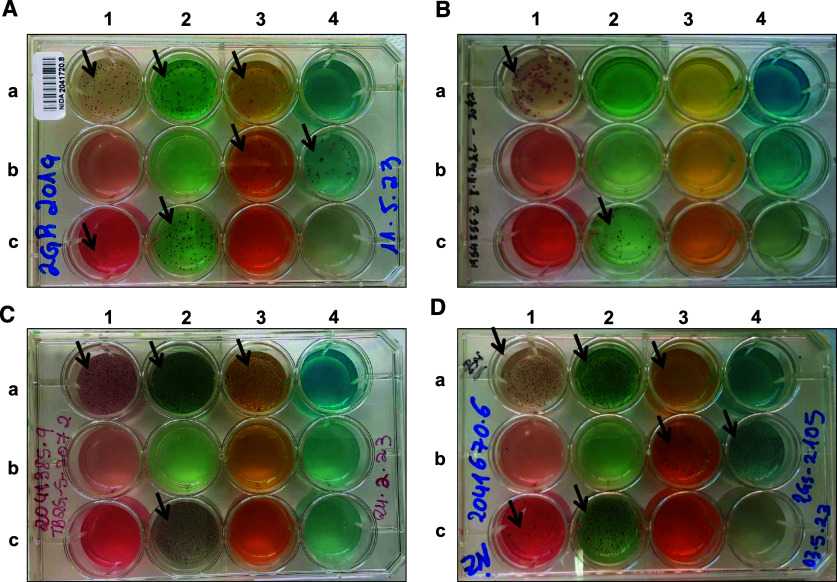
Interpretation of 2G test results obtained in the clinical setting using sputum samples from Xpert RIF susceptible and RIF resistant TB cases. **A:** The 2G test interpretation is growth of an MDR-*M.tb* complex strain with additional resistance to LNZ, AMK, PRO, and CYL. The sputum sample comes from a patient that is HIV negative with negative AFB; after microscopic evaluation, acid-fast bacilli growth on wells A1, A2, B4, and C2 lacks cord factor suggesting non-*M.tb* complex bacilli. The Xpert confirmatory result is ‘Trace’ (RIF resistance was confirmed with the Xpert diagnostic result of ‘Low’). No MGIT DST and LPA results were available. This is probably a mixed *M.tb* complex + NTM infection case. **B:** The 2G test interpretation is growth of an *M.tb* complex strain with monoresistance to CYL; the sputum sample comes from a patient that is HIV negative, with AFB 2+, an Xpert confirmatory result of ‘High’ with RIF susceptibility (no *rpoB* mutation). MGIT DST and LPA results were not available. **C:** The 2G test interpretation is growth of an MDR-*M.tb* complex strain with additional CYL resistance; the sputum sample comes from a patient that is HIV positive with AFB 2+. Growth on A1–A3 and C2 wells is compatible with acid-fast bacilli from *M.tb* complex showing cord factor in the microscopic evaluation. The confirmatory Xpert result is ‘High’ with RIF susceptibility (no *rpoB* mutation). MGIT result is *Mycobacterium tuberculosis* complex (MTBC), and MGIT DST shows additional resistance to INH and streptomycin. LPA result showed susceptibility to INH and RIF. This is an INH monoresistance detection by MGIT and the 2G test (missed by Xpert and LPA), with a potential RIF resistance detected by 2G but not confirmed by MGIT DST. **D:** The 2G test interpretation is growth of an MDR-*M.tb* complex strain with additional resistance to LNZ, AMK, PRO, and CYL. The sputum sample comes from a patient that is HIV negative with AFB 2+. Growth on A1, A2, B4, and C2 shows AFB lacking cord factor on the microscopy suggesting non-*M.tb* complex bacilli. Xpert confirmatory result is ‘Low’ with RIF susceptibility (no *rpoB* mutation). MGIT result is MTBC, but MGIT DST result was NTM, and LPA results showed susceptibility to INH and RIF. This is probably a mixed infection case, showing *M.tb* complex and NTM growth. MGIT result was initially MTBC, and when re-culturing for DST gave an NTM result.

Of the 18 2G test and MGIT DST positive results, 14 (78%) had valid results for the 2G test; of these 14 results, 9 arose from freshly processed sputum samples and 5 from frozen sputum samples. There were three RIF-resistant results (RR-TB) for the 2G test (using fresh sputa) not detected by either MGIT DST or Xpert. One MDR case detected by 2G test was RIF susceptible by Xpert, and INH- and streptomycin-resistant by MGIT DST. In addition, the 2G test detected one pre-XDR case (with resistance to AMK) that was MGIT DST contaminated and Xpert RIF resistant. The 2G test results from patients with DS and DR-*M.tb* complex are shown in Figure 4; as well as results showing mixed NTM and *M.tb* complex infection cases discussed in the figure legend.

## DISCUSSION

Here, we present the development of the first 2G test prototype and its performance in the laboratory and clinical settings. Our results in the laboratory using genotypically characterised DR-*M.tb* strains showed 100% sensitivity with phenotypic DST by the 2G test. Times to a positive result of the 2G test for interpretation were below 10 days when the 2G test was stored under optimal conditions (≤4 months at 4°C). These results are in concordance with our and others previous results using the 1G test (also called TB-CX test or colour test) that uses the same media and methodology but in a four-quadrant format to perform DST for INH, RIF, and an additional fluoroquinolone.^12–17^

In this clinical setting, the sensitivity of the 2G test compared to Xpert (45.9%) and MGIT (45.2%) culture was lower than expected compared to other published studies with the 1G test (TB-CX test) using solely fresh sputum samples, where sensitivity ranged from 65% to 80% when compared to Xpert and MGIT, respectively. Due to the logistical difficulties related to shipments during the COVID-19 pandemic (unknown humidity storage conditions at customs and during transit to the site), the 2G test readings might have yielded suboptimal results with regard to *M.tb* detection. Agar dehydration and decomposition of nutrient substrates might have played a role, probably driven by smaller volumes (∼3 mL) present in the wells of the 2G test 12-well format compared to the 4-well format of the 1G test (∼6 mL).^26^ Despite that the 2G test passed quality control (QC) before shipment and after receiving at the clinical setting, we cannot rule out that shipment and post-shipment storage conditions may have impaired growth of mycobacteria in the 2G test. These facts may explain some of the 2G test negative results obtained, and the poor sensitivity against Xpert and MGIT culture.^27^ Further studies should collect specific information on sputa as well as the 2G tests shipment conditions and storage upon receiving. A QC test using a known *M.tb* strain will need to be performed upon receiving, and another QC in parallel when testing samples. Comparison of the 2G test DST performance with MGIT DST was hindered by the high false-negative results of the 2G test; however, the 2G test detected three RR-TB that were not detected by either MGIT DST or Xpert. These could be due to *M.tb* strain(s) that carried a mutation conferring RIF resistance that could not be detected by Xpert^28–30^; however, we cannot rule out that these three RR-TB results could be false resistant results. Differences observed in the 2G test sensitivity when using fresh versus frozen samples compared to Xpert and MGIT may be due to the killing effect that sodium hydroxide (NaOH) has in *M.tb* complex survival after decontamination and also freeze/thaw cycles affecting *M.tb* viability specially if samples were somehow stored with traces of NaOH.^31^ Of note, we observed that the use of frozen sputum samples after digestion and decontamination with N-acetyl cysteine and NaOH provided results that were comparable or even better than MGIT for *M.tb* detection and DST in the 1G test.

## CONCLUSION

In the laboratory setting, the 2G test is a reliable diagnostic tool for detecting *M.tb* growth and performing DST simultaneously. However, in this clinical setting, the sensitivity of the 2G test to detect *M.tb* was lower than expected, being even lower when frozen instead of fresh sputa were used. This could be due to several reasons, including the storage conditions of the 2G test during shipment, the quality of the sputa, the need to perform a QC every time that a batch of sputa is being tested, and the lack of information on the samples to be tested related to their quality, transportation, and storage.

## Supplementary Material


