# Supplementary material for: Development and evaluation of the phenotypic 2G test to detect drug-resistant TB
Source: IJTLD Open. 2025 Nov 12;2(11):685–91. doi: 10.5588/ijtldopen.25.0326 (PMC12617084; doi:10.5588/ijtldopen.25.0326)
Supplement: Supplementary file 1 [file ijtldopen25-0326_supplementarydata1.pdf]

SUPLEMENTARY DATA

MATERIALS AND METHODS

Laboratory development of the 2G test prototype.

The 2G test was developed, manufactured, and quality control (QC) performed at Texas Biomedical Research Institute (Texas Biomed) in San Antonio, Texas, USA. The 2G test consists of a 12-well culture plate (Corning, Arizona, US), where 1 well has no drugs (drug susceptible detection, DS-well) and the rest of the wells contain different anti-TB drugs at their CC as defined by WHO (1) (**Fig 1**). The medium for the 2G test was prepared with a combination of 7H11 agar (Remel agar, Thermo Fisher Scientific, Waltham, MA) specific for mycobacterial growth, carbendazim (a fungicide), and glycerol that was all mixed and autoclaved at 121°C with a 10-min sterilization period. Once the agar medium was cooled to 56°C, oleic acid albumin dextrose complex (OADC), Selectatab (Kirchner, Mast Laboratories Ltd, Merseyside, UK), and 2,3-Diphenyl-5-thienyl-(2)-tetrazolium chloride (STC) (TCI Europe, Zwijndrecht, Belgium) were added. Selectatab is a selective supplement used to suppress the growth of common sputum contaminants. The STC is an oxidation-reduction indicator that allows colonies to grow red and be visible to the naked eye as previously described (2-6). Anti-TB drugs without an established CC in 7H11 were added to the agar to reach the following concentrations: pyrazinamide (PZA) at 1,100 μg/mL, amikacin (AMK), at 2 μg/mL, protionamide (PRO) at 10 μg/mL, cycloserine (CYL) at 32 μg/mL, and clofazimine (CLO) at 1 μg/mL. The selection of the CC for PZA was based in the following research paper (7). The selected CC of 2 μg/mL for AMK was used as a proxy as it is the CC recommended for 7H10 agar media (1). The CC of 10 μg/mL for PRO was used as a proxy as it is the CC for ethionamide in 7H11 agar medium; the CC of 32 μg/mL selected for CYL was based in the following research paper (8), and the selected CC for CLO was based on its CC established in liquid culture (1). Combinations of food colorants were used to distinguish wells with the different anti-TB drugs.

2G test shipment to CISM

Twelve lots of the 2G test prototype were prepared by Texas Biomed and shipped to Manhica, Southern Mozambique from July 2021 to June 2023. The shipment lot was laboratory validated (pre-shipment QC) using known DS- and DR-*M.tb* strains and stored at 4°C until shipment to the field site. A post-shipment DST QC check was also performed upon arrival at the study site. Only lots that passed this second QC were used. Shipment was performed at room temperature using

a commercial courier. The shipment time to reach our testing site in Mozambique (transit time) oscillated between 3 to 8 weeks due to the COVID-19 pandemic and unforeseen logistical issues with Mozambique customs. During this transit time, the storage conditions (temperature/humidity) of 2G tests under Mozambique authorities are unknown. Once the 2G test prototype cargo was cleared from Mozambique customs, 2G tests were stored at 4°C or in some cases at room temperature until their use. The shelf life of the 2G test prototypes is about ~3 months when stored at both 4°C and/or room temperature.

Xpert MTB/RIF Ultra (Xpert) test at CISM – Raw sputum (2 mL) was mixed with sodium hydroxide and isopropanol-containing sample reagent (SR; Cepheid, Sunnyvale, CA) in proportion 1:2 (v/v). The mixture was incubated for 15 min with occasional shaking and then added to the sample loading chamber of the cartridge for automatic processing in the Xpert platform following the manufacturer instructions (9).

Mycobacteria Growth Indicator Tube (MGIT) liquid culture and DST at CISM– Sputum samples were decontaminated by the modified Kubica method (10). Afterwards, 500 μL were inoculated into MGIT liquid culture and incubated in the BACTEC MGIT 960 instrument according to manufacturer´s guidelines. After 42 days of culture without growth, samples were classified as negative. Time to a positive result was defined as the number of days from MGIT inoculation to the positive signal detected by BACTEC MGIT 960 instrument. An immunochromatographic rapid assay was performed to confirm the presence of *M.tb* complex (SD Bioline TB Ag MPT64 assay (Standard Diagnostics, Inc., Korea), and blood agar and AFB were used to verify the culture purity. Positive MGIT cultures were sub-cultured to assess their sensitivity to streptomycin (STM), INH, RIF, and ethambutol (EMB) following manufacturer's recommendations (11). Drug concentrations in MGIT DST were set at 1.0 µg/mL for STM, 0.1 µg/mL for INH, 1.0 µg/mL for RIF, and1.0 µg/mL for EMB.

The 2G test procedure at CISM– The 2G tests were shipped to CISM. The 2G tests were inoculated with fresh or frozen stored samples, incubated at 37°C for a minimum of 3 days prior to the first reading, and subsequently, read every 3 days until visible colony forming units (CFUs) were observed in the DS-well or for up to 42 days. The DS-well was read using a 40X magnifier glass (AIXPI 40X magnifier, Shenzhen, China). *M.tb* complex colony growth was characterized via visible rough red colonies due to the STC compound in the medium. We used the same interpretation parameters as published for the established 1G test (TB-CX test) (2-6, 12). Days to

a positive result for the 2G test prototype was defined as the number of days passed since inoculum until a positive growth (growth with countable CFUs) is observed in the DS-well. The 2G test follows the agar proportion method with the 1% critical proportion to determine susceptibility or resistance to a certain drug (13, 14).

RESULTS

Table 1: Patients' socio-demographic and clinical characteristics at enrollment

Variable	Variable value	n (sample size)	n (%) / IQR*
**Sex**		**138**	
	Female	56	40.6
**Age**		**138**	
			38.8 (31 - 52.3)
**Recruitment site**		**137**	
	Manhiça	84	61.31
	Xinavane	16	11.68
	Magude	8	5.84
	Others	29	21.17
**Contact with a TB patient**		**138**	
	Yes	9	6.52
**Smoking**		**138**	
	Yes	4	2.90
**TB type**		**138**	
	New	129	93.48
**HIV status**		**137**	
	Positive	47	34.31
**ART**		**46**	97.80
	Yes	36	78.26
**Cotrimoxazole prophylaxis**		**46**	97.80
	Yes	34	73.91
**TB site**		**137**	
	Pulmonary	137	100.00
**TB symptoms**			
Cough		**138**	
	Yes	137	99.3
Cough duration			21 (15 - 30)
Fever		**137**	
	Yes	101	73.72
Fever duration			15 (15 - 21)
Weight loss		**135**	

	Yes	87	64.44
Weight loss duration			15 (15 - 21)
Night sweats		**137**	
	Yes	96	70.07
Night sweats duration			15 (15 - 21)

* IQR for continuous variables only

REFERENCES

1. World Health Organization. Technical manual for drug susceptibility testing of medicines used in the treatment of tuberculosis. Geneva: World Health Organization; 2018 2018. Report No.: 9789241514842.
2. Klaos K, Agejeva A, Kummik T, Laks S, Remets O, Sasi S, et al. A successful introduction to a non-expert setting of the thin-layer agar Colour Test as an indirect phenotypic drug susceptibility test for Mycobacterium tuberculosis. International Journal of Infectious Diseases. 2021;104:19-26.
3. Shibabaw A, Gelaw B, Kelley H, Balada-Llasat JM, Evans C, Wang SH, et al. Accuracy of the color plate micro-colony detection for the diagnosis of Mycobacterium tuberculosis complex in Northwest Ethiopia. Tuberculosis. 2019;114(March 2018):54--60.
4. Shibabaw A, Gelaw B, Kelley HV, Tesfaye E, Balada-Llasat JM, Evans CA, et al. MDR/XDR-TB Colour Test for drug susceptibility testing of Mycobacterium tuberculosis, Northwest Ethiopia. Int J Infect Dis. 2020;90:213-8.
5. Toit K, Mitchell S, Balabanova Y, Evans CA, Kummik T, Nikolayevskyy V, et al. The Colour Test for drug susceptibility testing of Mycobacterium tuberculosis strains. Int J Tuberc Lung Dis. 2012;16(8):1113-8.
6. Zhang A, Jumbe E, Krysiak R, Sidiki S, Kelley HV, Chemey EK, et al. Low-cost diagnostic test for susceptible and drug-resistant tuberculosis in rural Malawi. Afr J Lab Med. 2018;7(1):690.
7. Heifets L, Sanchez T. New agar medium for testing susceptibility of Mycobacterium tuberculosis to pyrazinamide. J Clin Microbiol. 2000;38(4):1498-501.
8. Yu X, Zeng X, Shi W, Hu Y, Nie W, Chu N, et al. Validation of Cycloserine Efficacy in Treatment of Multidrug-Resistant and Extensively Drug-Resistant Tuberculosis in Beijing, China. Antimicrob Agents Chemother. 2018;62(3).
9. Cepheid. Xpert® MTB/RIF Ultra Instructions for Use. 302-5776, Rev. D ed: Cepheid; 2023.
10. Kubica GP, Dye WE, Cohn ML, Middlebrook G. Sputum digestion and decontamination with N-acetyl-L-cysteine-sodium hydroxide for culture of mycobacteria. Am Rev Respir Dis. 1963;87:775-9.
11. Becton Dickinson. BD BACTEC™ MGIT™ 960 SIRE Kits For the Antimycobacterial Susceptibility Testing of Mycobacterium tuberculosis 2019 [Available from: https://www.bd.com/resource.aspx?IDX=18269.
12. Mekonnen B, Mihret A, Getahun M, Hailu T, Sidiki S, H VK, et al. Evaluation of the tuberculosis culture color plate test for rapid detection of drug susceptible and drug-resistant Mycobacterium tuberculosis in a resource-limited setting, Addis Ababa, Ethiopia. PLoS One. 2019;14(5):e0215679.

13. World Health Organization. Technical report on critical concentrations for drug susceptibility testing of isoniazid and the rifamycins (rifampicin, rifabutin and rifapentine). Geneva: World Health Organization; 2021 2021.
14. Canetti G, Froman S, Grosset J, Hauduroy P, Langerova M, Mahler HT, et al. MYCOBACTERIA: LABORATORY METHODS FOR TESTING DRUG SENSITIVITY AND RESISTANCE. Bull World Health Organ. 1963;29(5):565-78.
